# Geographic Variation in the Association between Ambient Fine Particulate Matter (PM_2.5_) and Term Low Birth Weight in the United States

**DOI:** 10.1289/ehp.1408798

**Published:** 2015-06-05

**Authors:** Yongping Hao, Heather Strosnider, Lina Balluz, Judith R. Qualters

**Affiliations:** National Center for Environmental Health, Centers for Disease Control and Prevention, Atlanta, Georgia, USA

## Abstract

**Background:**

Studies on the association between prenatal exposure to fine particulate matter ≤ 2.5 μm in aerodynamic diameter (PM_2.5_) and term low birth weight (LBW) have resulted in inconsistent findings. Most studies were conducted in snapshots of small geographic areas and no national study exists.

**Objectives:**

We investigated geographic variation in the associations between ambient PM_2.5_ during pregnancy and term LBW in the contiguous United States.

**Methods:**

A total of 3,389,450 term singleton births in 2002 (37–44 weeks gestational age and birth weight of 1,000–5,500 g) were linked to daily PM_2.5_ via imputed birth days. We generated average daily PM_2.5_ during the entire pregnancy and each trimester. Multi-level logistic regression models with county-level random effects were used to evaluate the associations between term LBW and PM_2.5_ during pregnancy.

**Results:**

Without adjusting for covariates, the odds of term LBW increased 2% [odds ratio (OR) = 1.02; 95% CI: 1.00, 1.03] for every 5-μg/m^3^ increase in PM_2.5_ exposure during the second trimester only, which remained unchanged after adjusting for county-level poverty (OR = 1.02; 95% CI: 1.01, 1.04). The odds did change to null after adjusting for individual-level predictors (OR = 1.00; 95% CI: 0.99, 1.02). Multi-level analyses, stratified by census division, revealed significant positive associations of term LBW and PM_2.5_ exposure (during the entire pregnancy or a specific trimester) in three census divisions of the United States: Middle Atlantic, East North Central, and West North Central, and significant negative association in the Mountain division.

**Conclusions:**

Our study provided additional evidence on the associations between PM_2.5_ exposure during pregnancy and term LBW from a national perspective. The magnitude and direction of the estimated associations between PM_2.5_ exposure and term LBW varied by geographic locations in the United States.

**Citation:**

Hao Y, Strosnider H, Balluz L, Qualters JR. 2016. Geographic variation in the association between ambient fine particulate matter (PM_2.5_) and term low birth weight in the United States. Environ Health Perspect 124:250–255; http://dx.doi.org/10.1289/ehp.1408798

## Introduction

Low birth weight (LBW) is a known risk factor for infant morbidity and mortality and chronic health problems in later life (McCormick 1985). Maternal exposure to particulate matter (PM_2.5_—fine particulate matter with aerodynamic diameter ≤ 2.5 μm; and PM_10_—particulate matter with aerodynamic diameter ≤ 10 μm) during pregnancy may contribute to adverse reproductive health outcomes including term LBW (Backes et al. 2013; Dadvand et al. 2013; Fleischer et al. 2014; Pedersen et al. 2013; Sapkota et al. 2012; Stieb et al. 2012). Findings from studies of associations between prenatal exposure to PM_2.5_ and PM_10_ and adverse reproductive health outcomes have been inconsistent (Bosetti et al. 2010; Sapkota et al. 2012; Shah and Balkhair 2011; Stieb et al. 2012). For instance, PM_10_ and PM_2.5_ were found to be associated with term LBW in Connecticut and Massachusetts (Bell et al. 2007b), California and six northeastern cities (PM_2.5_) (Maisonet et al. 2001; Parker et al. 2005), Allegheny County, Pennsylvania (PM_10_) (Xu et al. 2011), United States; Europe (PM_2.5_) (Pedersen et al. 2013); São Paulo, Brazil (PM_10_) (Gouveia et al. 2004); and Seoul, Korea (PM_10_) (Lee et al. 2003); however, no such evidence was reported in Seattle, Washington, United States (PM_2.5_) (Dadvand et al. 2013); Oslo, Norway (Madsen et al. 2010); or the Netherlands (Gehring et al. 2011; Pedersen et al. 2013); and mixed evidence exists in a few systematic reviews and meta-analyses (Sapkota et al. 2012; Shah and Balkhair 2011; Stieb et al. 2012). Heterogeneity in the published findings may arise from differences in many aspects of the study designs and available data. For example, the methods of assigning exposure may vary, given that the consistent and high quality air pollution exposure data were rarely available across large geographic areas in the past.

Most published studies have limited geographic areas or time periods, often with small sample size, in part due to sparsely distributed air pollution monitoring data. A few studies with larger geographic coverage have reported geographic variation in associations between air pollution and LBW. A study of term singleton births from 397 counties in the United States showed that the associations between PM_2.5_ and term LBW varied greatly by region (Parker and Woodruff 2008). A recent international collaboration reexamined data from multiple countries using a standard protocol and confirmed the existence of geographical variation in associations between PM_2.5_ and LBW (Dadvand et al. 2013). To our best knowledge, no national study in the United States has previously linked daily PM_2.5_ with gestational ages of pregnancies and examined the associations between PM_2.5_ exposure during pregnancy and term LBW. In this study, we linked 2001–2002 daily PM_2.5_ estimates with all term singleton births (3,389,450) in 2002 in all 3,109 counties in the contiguous United States and explored geographic variation in the associations between ambient PM_2.5_ exposure and term LBW via a multilevel approach. We limited our study area to the contiguous United States (48 states and District of Columbia), because PM_2.5_ data are not available for the noncontiguous states of Alaska and Hawaii.

## Methods

*Study population.* Birth data used in this study were obtained from the National Environmental Public Health Tracking Network (Tracking Network) [Centers for Disease Control and Prevention (CDC) 2013]. The Tracking Network is a system of integrated health, exposure, and hazard information and data from a variety of national, state, and city sources. Birth data on the Tracking Network were obtained from the National Center for Health Statistics (NCHS), CDC (Martin et al. 2007). We included all singletons with gestational age of 37–44 weeks and birth weight of 1,000 g–5,500 g (3,389,450 term births) born to mothers who resided in the contiguous United States in 2002. The U.S. birth certificate system underwent a revision starting 2003; states have implemented this system in a piecemeal manner over the years (ftp://ftp.cdc.gov/pub/Health_Statistics/NCHS/Dataset_Documentation/DVS/natality). To avoid the data-coding inconsistency problems associated with birth certificate revision, we decided to use 2002 birth data only. We excluded those births with missing values of race/ethnicity (18,215; < 1%), parity (6,960; < 1%), maternal education (41,098; 1%), and prenatal care utilization (55,663; 2%). We further excluded all births with missing data for smoking status (14,036; < 1%) except for California births, because smoking status was not recorded in California birth certificates in 2002, and an unknown category of smoking status was assigned for all California births. Our final analytical data set included 3,271,203 (96.5%) term births.

*PM_2.5_ exposure assignment.* The 2001–2002 daily census tract–level PM_2.5_ concentration data for the contiguous United States were generated by the U.S. Environmental Protection Agency (EPA) for the Tracking Network using hierarchical Bayesian models based on data from the Community Multi-scale Air Quality modeling system, including emission, meteorology, and chemical modeling components and air monitoring stations (McMillan et al. 2010; U.S. EPA 2010). Census tract is the geographic unit nested to county and is often used as a geographic proxy for local community. On average, a census tract contains about 4,300 inhabitants, ranging from 0 to 36,146 in 2000. We aggregated census tract–level daily PM_2.5_ estimates and weighted by 2000 Census tract populations to generate county-level daily PM_2.5_ estimates for 3,109 counties (average 89,927 inhabitants, ranging from 67 to 9,519,338 in 2000) in the contiguous United States.

We linked birth data and daily PM_2.5_ estimates by both county identifiers and pregnancy dates from conception to birth. The start and end date of pregnancy or trimester were determined by infant birth date and gestational age (available only in completed weeks) at birth. Because only birth month, instead of birth date, was accessible to researchers due to confidentiality, we imputed birth day as the random day within the birth month via a uniform distribution, which means that any day within the birth month could be a birth day with equal probability. Individualized PM_2.5_ exposure of a term birth was summarized as county-level average daily PM_2.5_ concentration during the entire pregnancy and each trimester (first trimester: weeks 1–13; second trimester: weeks 14–26; and third trimester: weeks 27–birth or 44 weeks) based on the maternal county of residence listed on the birth certificate. Thus, each birth had PM_2.5_ exposure estimates for the entire pregnancy, and for the first, second, and third trimesters.

*Main outcome and covariates.* Our outcome variable was term LBW (1,000 g < weight < 2,500 g), versus normal birth weight (2,500 g ≤ weight < 5,500 g). Individual-level predictors included average daily PM_2.5_ as well as infant and maternal demographics. Average daily PM_2.5_ during the entire pregnancy and each trimester for each birth was treated as a continuous predictor. All other predictors were categorical: infant sex (female vs. male), parity (first live birth vs. non-first live birth), gestational age (37, 38, 39, and 40–44 weeks), maternal age (< 20, 20–34, and ≥ 35 years), maternal race/ethnicity (Hispanic, non-Hispanic white, non-Hispanic black, and other races), marital status (not married vs. married), educational attainment (< 12, 12, 13–15, and ≥ 16 years), prenatal care start time (fourth month or later/no care vs. first–third month), smoking (smoker, nonsmoker), birth season [spring (March–May), summer (June–August), fall (September–November), and winter (December–February)]; and nine U.S. census divisions (New England, Middle Atlantic, East North Central, West North Central, South Atlantic, East South Central, West South Central, Mountain, Pacific) (U.S. Census Bureau 2005).

Our county-level socioeconomic status (SES) predictor was poverty, measured as the percentage of county residents below the federal poverty line. Prior studies reveal that this measure is superior to other area-based measures of SES (e.g., median home value) in sensitivity to SES-related health outcomes (Krieger 2007). We obtained 2002 county poverty data from U.S. Census’ Small Area Income and Poverty Estimates program (U.S. Census Bureau 2013) and linked them with birth data. Counties were categorized into four groups according to their poverty rates: < 10%, 10%–14.9%, 15%–19.9%, and ≥ 20%.

*Statistical analysis.* We used multilevel logistic regression models with county-level random effects to examine the associations of PM_2.5_ exposure during pregnancy and term LBW. The odds ratio (OR) or adjusted odds ratio (AOR) was used to measure associations. A *p*-value < 0.05 was used to define statistical significance of associations.

First, we conducted the main analyses using full study population with four different PM_2.5_ exposure estimates: entire pregnancy, first trimester, second trimester, and third trimester. A series of models were constructed for each of four PM_2.5_ exposure estimates during pregnancy, and three of them are presented: model 1 included only PM_2.5_ exposure and county-level random effects; model 2 was model 1 plus county poverty; and model 3 was model 2 plus all individual predictors, including infant’s sex and parity, gestational age, mother’s age, race/ethnicity, marital status, education, prenatal care, birth season, and census division. We presented AORs (OR for model 1) and confidence intervals (CIs) for independent variables while accounting for potential within-county correlations among term births from the same counties via county-level random effects.

Second, we conducted stratified analyses by U.S. census division to explore the associations between term LBW and PM_2.5_ exposure during pregnancy, adjusting for both individual- and county-level predictors. The U.S. census division map is available at http://www2.census.gov/geo/pdfs/maps-data/maps/reference/us_regdiv.pdf.SAS PROC GLIMMIX (SAS Institute Inc.) was used to implement the multilevel logistic models in this study, accounting for county-level random effects. Given the narrow range of PM_2.5_ exposure, we present AORs for every 5-μg/m^3^ increase in PM_2.5_ exposure.

Finally, we conducted two sensitivity analyses. The first sensitivity analysis was designed to evaluate whether including smoking in the models had any impact on the results from the main and stratified analyses. The second sensitivity analysis was to link U.S. EPA monitor-based PM_2.5_ estimates with 2,435,805 births from 687 counties in 48 states and the District of Columbia and to repeat the main and stratified analyses.

## Results

*Characteristics of term singleton births.* Overall, there were 3,271,203 eligible term singleton infants in the contiguous United States, and 81,797 (3%) of them were term LBW infants in 2002 (Table 1). The proportion of term LBW infants was highest among female infants (3%), first live births (3%), births with gestational age of 37 weeks (8%), births to mothers with maternal age of < 20 years (4%), non-Hispanic black mothers (5%), unmarried mothers (4%), mothers with < 12 years of education (3%), mothers with delayed or no prenatal care (3%), mothers who are smokers (5%), and mothers residing in counties with poverty rates of 20% or higher (3%) compared with their counterparts (Table 1).

**Table 1:** Selected characteristics of singleton term births (37–44 weeks gestational age), United States, 2002.

Characteristic	Term births *n* (%)	Term LBW *n* (%)^*a*^
USA (48 states and District of Columbia)	3,271,203 (100)	81,797 (3)
Infant sex
Female	1,606,780 (49)	47,850 (3)
Male	1,664,423 (51)	33,947 (2)
Parity
First live birth	1,320,923 (40)	40,219 (3)
Non-first live birth	1,950,280 (60)	41,578 (2)
Gestational age (weeks)
37	312,366 (10)	25,193 (8)
38	660,074 (20)	22,049 (3)
39	930,241 (28)	16,222 (2)
40–44	1,368,522 (42)	18,333 (1)
Maternal age (years)
< 20	343,361 (10)	13,235 (4)
20–34	2,489,078 (76)	58,262 (2)
≥ 35	438,764 (13)	10,300 (2)
Maternal race/ethnicity
Non-Hispanic white	1,928,006 (59)	39,106 (2)
Non-Hispanic black	441,860 (14)	20,657 (5)
Hispanic	711,217 (22)	16,296 (2)
Other race	190,120 (6)	5,738 (3)
Marital status
Not married	1,071,672 (33)	39,318 (4)
Married	2,199,531 (67)	42,479 (2)
Maternal education attainment (years)
< 12	689,264 (21)	23,829 (3)
12	1,006,961 (31)	28,845 (3)
13–15	706,542 (22)	15,409 (2)
≥ 16	868,436 (27)	13,714 (2)
Prenatal care start time (month)
Fourth month or later/no care	509,866 (16)	17,603 (3)
First–third month	2,761,337 (84)	64,194 (2)
Smoking status
Unknown^*b*^	421,172 (13)	8,594 (2)
Smoker	316,727 (10)	17,103 (5)
Non-smoker	2,533,304 (77)	56,100 (2)
Birth season^*c*^
Spring	808,268 (25)	19,740 (2)
Summer	850,268 (26)	21,178 (2)
Fall	828,255 (25)	20,819 (3)
Winter	784,412 (24)	20,060 (3)
County poverty rate^*d*^
≥ 20%	239,503 (7)	7,982 (3)
15%–19.9%	609,592 (19)	17,447 (3)
10%–14.9%	1,304,935 (40)	33,439 (3)
< 10%	1,117,173 (34)	22,929 (2)
Census division
New England	142,575 (4)	3,017 (2)
Middle Atlantic	413,520 (13)	9,972 (2)
East North Central	507,796 (16)	12,623 (2)
West North Central	218,318 (7)	4,525 (2)
South Atlantic	599,403 (18)	16,972 (3)
East South Central	188,987 (6)	5,984 (3)
West South Central	431,798 (13)	12,252 (3)
Mountain	250,368 (8)	6,230 (2)
Pacific	518,438 (16)	10,222 (2)
LBW, low birth weight. ^***a***^The percentage of term LBW was obtained by dividing the number of term LBW by the number of term births in the corresponding category. ^***b***^Unknown category exclusively reflects California singleton term births–smoking status was not recorded in California birth certificate for 2002. ^***c***^Birth season: spring (March–May), summer (June–August), fall (September–November), and winter (December–February); birth season is the only variable that was not significantly associated with term LBW. ^***d***^The percentage of people in a county below the federal poverty line.		

*PM_2.5_ exposure during pregnancy.* Average daily PM_2.5_ exposures ranged from 4.7 μg/m^3^ to 23.8 μg/m^3^ for the entire pregnancy, and from a minimum of 3.3 μg/m^3^ to a maximum of 30.1 μg/m^3^ during individual trimesters (Table 2). Average daily PM_2.5_ exposure during the entire pregnancy was strongly correlated with trimester-specific PM_2.5_ exposures (Pearson correlation coefficients 0.81–0.86), but the correlations among trimester-specific PM_2.5_ exposures were weaker (0.46–0.59). Table 2 shows the PM_2.5_ exposure during the entire pregnancy and three trimesters for each census division. Average PM_2.5_ exposure during pregnancy was generally highest (> 13 μg/m^3^) in Middle Atlantic, East North Central, and Pacific divisions and lowest (< 10 μg/m^3^) in the Mountain division.

**Table 2:** County-level average daily PM_2.5_ (μg/m^3^) exposure during pregnancy by census division (*n *= 3,271,203).

Location	Entire pregnancy mean (min, max), IQR	Trimester		
		First mean (min, max), IQR	Second mean (min, max), IQR	Third mean (min, max), IQR
USA (48 states and District of Columbia)	12.5 (4.7, 23.8), 4.1	12.5 (3.7, 29.6), 4.4	12.6 (3.6, 29.6), 4.4	12.6 (3.3, 30.1), 4.6
Census division
New England	11.8 (7.0, 14.5), 1.8	11.8 (6.2, 16.5), 2.5	11.9 (6.2, 17.4), 2.8	11.6 (5.5, 18.5), 3.2
Middle Atlantic	13.7 (7.3, 18.6), 2.5	13.6 (6.4, 24.6), 3.2	13.9 (6.4, 24.6), 3.7	13.6 (6.0, 25.2), 3.8
East North Central	13.8 (6.5, 18.1), 2.3	13.6 (5.8, 23.4), 2.8	13.7 (5.8, 25.2), 2.7	14.1 (5.6, 25.7), 3.1
West North Central	10.5 (5.7, 16.3), 2.0	10.4 (5.3, 18.1), 2.0	10.5 (5.3, 21.0), 2.2	10.7 (4.7, 21.2), 2.4
South Atlantic	12.2 (4.7, 18.1), 4.0	12.4 (3.9, 24.0), 4.0	12.2 (3.6, 23.2), 4.0	12.1 (3.3, 24.3), 4.0
East South Central	13.0 (9.3, 18.0), 2.3	13.1 (7.9, 23.2), 3.9	13.1 (7.8, 25.0), 3.7	12.9 (7.9, 25.5), 3.8
West South Central	10.8 (5.6, 15.0), 2.6	10.6 (5.2, 18.4), 3.0	10.8 (5.2, 18.4), 3.0	10.9 (4.3, 18.4), 3.1
Mountain	9.0 (5.0, 14.6), 2.6	8.9 (4.0, 25.4), 2.5	9.1 (4.1, 25.4), 2.4	9.0 (3.7, 28.4), 2.6
Pacific	14.9 (4.8, 23.8), 9.1	14.9 (3.7, 29.6), 10.1	14.8 (3.8, 29.6), 9.2	14.9 (3.9, 30.1), 8.7
Abbreviations: IQR, interquartile range; max, maximum; min, minimum.				

*Multilevel models for the contiguous United States.* Associations between PM_2.5_ exposure and term LBW differed for exposures averaged over the entire pregnancy and individual trimesters (Table 3). Before adjusting for covariates (model 1), the OR for LBW in association with a 5-μg/m^3^ increase in PM_2.5_ during the second trimester was 1.02 (95% CI: 1.00, 1.03). The OR (OR = 1.02; 95% CI: 1.01, 1.04) was similar after adjusting for county-level poverty (model 2), but null (OR = 1.00; 95% CI: 0.99, 1.02) after additionally adjusting for individual-level predictors (model 3). There was a nonsignificant positive association between term LBW and PM_2.5_ exposure during the entire pregnancy based on model 2 (OR = 1.02; 95% CI: 0.99, 1.05) but not model 3 (OR = 0.99; 95% CI: 0.96, 1.02). We estimated nonsignificant negative associations with exposure during the third trimester based on all three models (e.g., OR = 0.99; 95% CI: 0.97, 1.00 for model 3). Analyses stratified by county poverty levels did not show consistent patterns, though LBW was significantly increased in association with PM_2.5_ exposure over the entire pregnancy in counties with the highest poverty rates (≥ 20%) (OR = 1.11; 95% CI: 1.00, 1.22) (see Supplemental Material, Table S1).

**Table 3:** Odds ratio of term LBW associated with average daily PM_2.5_ exposure during pregnancy in the contiguous United States.*^a^*

Trimester	Model 1^*b*^[OR (95% CI)]	Model 2^*c*^[AOR (95% CI)]	Model 3^*d*^[AOR (95% CI)]
*n*	3,271,203	3,271,203	3,271,203
Entire pregnancy	0.99 (0.96, 1.02)	1.02 (0.99, 1.05)	0.99 (0.96, 1.02)
First trimester	0.99 (0.97, 1.01)	1.00 (0.98, 1.01)	1.00 (0.99, 1.02)
Second trimester	1.02 (1.00, 1.03)*	1.02 (1.01, 1.04)*	1.00 (0.99, 1.02)
Third trimester	0.99 (0.97, 1.00)	0.99 (0.98, 1.01)	0.99 (0.97, 1.00)
Abbreviations:**AOR, adjusted odds ratios; CI, confidence intervals; LBW, low birth weight; OR, odds ratios. ^***a***^Effect estimates (95% CI) are reported as per 5-μg/m^3^ increase in PM_2.5_. ^***b***^Model 1: PM_2.5_ + county-level random effects. ^***c***^Model 2: model 1 + county-level poverty. ^***d***^Model 3: model 2 + individual-level covariates, including infant’s sex and parity, gestational age, mother’s age, race/ethnicity, marital status, education, prenatal care, birth season, and census division. *p < 0.05.			

**Figure 1:**
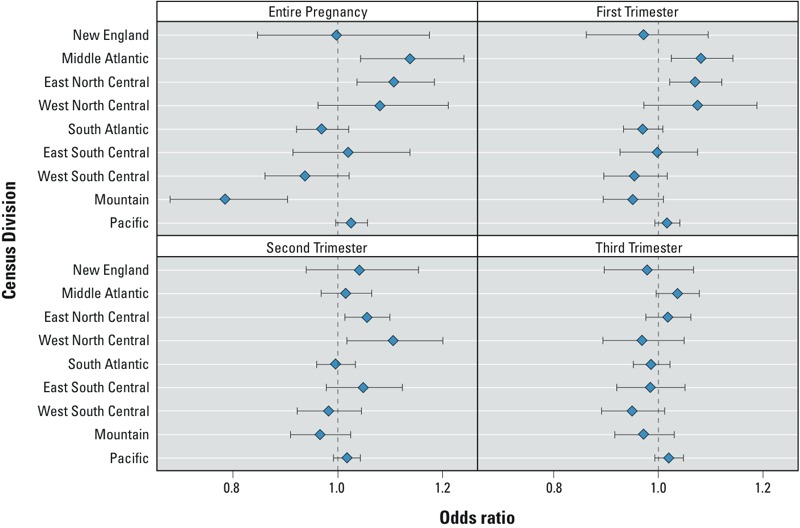
Adjusted odds ratio of term LBW associated with average daily PM_2.5_ exposure by census division during entire pregnancy, first trimester, second trimester, and third trimester. All models included average daily PM_2.5_ exposure estimate for entire pregnancy or individual trimester, individual-level covariates: infant’s sex and parity, gestational age, mother’s age, race/ethnicity, marital status, education, prenatal care, birth season, and county-level poverty rate and county-level random effects.

*Multilevel models by census division.* Multilevel models stratified by census division (Figure 1, Table 4) showed that, after adjusting for individual- and county-level variables, the AOR between PM_2.5_ exposure and term LBW differed by census division. Significant positive associations between LBW and PM_2.5_ exposure were estimated for three census divisions: the Middle Atlantic (during the entire pregnancy, OR = 1.14; 95% CI: 1.04, 1.24; and the first trimester, OR = 1.08; 95% CI: 1.03, 1.14); East North Central (during the entire pregnancy and the first and second trimesters, e.g., entire pregnancy OR = 1.11; 95% CI: 1.04, 1.18); and West North Central divisions (second trimester OR = 1.11; 95% CI: 1.02, 1.20). There was a significant negative association between PM_2.5_ exposure over the entire pregnancy and LBW in the Mountain division (OR = 0.78; 95% CI: 0.68, 0.90).

**Table 4:** Adjusted odds ratio of term LBW associated with average daily PM_2.5_ exposure during pregnancy by census division.*^a^*

Census division	*n*	Entire pregnancy [AOR (95% CI)]	First trimester [AOR (95% CI)]	Second trimester [AOR (95% CI)]	Third trimester [AOR (95% CI)]
New England	142,575	1.00 (0.85, 1.17)	0.97 (0.86, 1.10)	1.04 (0.94, 1.15)	0.98 (0.90, 1.07)
Middle Atlantic	413,520	1.14 (1.04, 1.24)*	1.08 (1.03, 1.14)*	1.02 (0.97, 1.06)	1.04 (1.00, 1.08)
East North Central	507,796	1.11 (1.04, 1.18)*	1.07 (1.02, 1.12)*	1.06 (1.01, 1.10)*	1.02 (0.98, 1.06)
West North Central	218,318	1.08 (0.96, 1.21)	1.08 (0.97, 1.19)	1.11 (1.02, 1.20)*	0.97 (0.89, 1.05)
South Atlantic	599,403	0.97 (0.92, 1.02)	0.97 (0.93, 1.01)	1.00 (0.96, 1.03)	0.99 (0.95, 1.02)
East South Central	188,987	1.02 (0.91, 1.14)	1.00 (0.93, 1.08)	1.05 (0.98, 1.12)	0.98 (0.92, 1.05)
West South Central	431,798	0.94 (0.86, 1.02)	0.95 (0.90, 1.02)	0.98 (0.92, 1.05)	0.95 (0.89, 1.01)
Mountain	250,368	0.78 (0.68, 0.90)*	0.95 (0.90, 1.01)	0.97 (0.91, 1.02)	0.97 (0.92, 1.03)
Pacific	518,438	1.03 (1.00, 1.06)	1.02 (0.99, 1.04)	1.02 (0.99, 1.04)	1.02 (0.99, 1.05)
California	421,721	1.02 (0.99, 1.06)	1.01 (0.99, 1.04)	1.02 (0.99, 1.05)	1.02 (0.99, 1.06)
Abbreviations: AOR, adjusted odds ratios; CI, confidence intervals; LBW, low birth weight. ^***a***^Effect estimates (95% CI) are reported as per 5-μg/m^3^ increase in PM_2.5_; all models include county-level random effects, PM_2.5_ exposure during either entire pregnancy or a specific trimester, county-level poverty rate, and other individual-level covariates: infant’s sex and parity, gestational age, mother’s age, race/ethnicity, marital status, education, prenatal care, and birth season. *p < 0.05.					

*Sensitivity analyses.* The analyses that adjusted smoking status yielded almost the same results (see Supplemental Material, Tables S2 and S3) as our main and stratified analyses (Tables 3 and 4), suggesting that the exclusion of maternal smoking status had little impact on the main results. The sensitivity analyses using monitor-based PM_2.5_ estimates generated similar results for the main analyses, except for a significant positive association for PM_2.5_ over the entire pregnancy based on model 1 (OR = 1.04; 95% CI: 1.01, 1.07) (see Supplemental Material, Table S4). The sensitivity analyses using monitor-based PM_2.5_ estimates also generated similar results for the stratified analyses by census division (see Supplemental Material, Table S5). Significant positive associations between LBW and PM_2.5_ exposure were also estimated for two census divisions: the Middle Atlantic (first trimester, OR = 1.06; 95% CI: 1.00, 1.11); and West North Central divisions (second trimester OR = 1.12; 95% CI: 1.01, 1.24). There was also a significant negative association between PM_2.5_ exposure over the entire pregnancy and LBW in the Mountain division (OR = 0.90; 95% CI: 0.81, 1.00).

## Discussion

To our knowledge, this is the first national study that linked daily PM_2.5_ with individual gestational ages of term births in the contiguous United States to examine the associations between term LBW and PM_2.5_ exposure during the entire pregnancy as well as during specific trimesters (first, second, and third) in a multilevel framework. We used highly resolved PM_2.5_ data to estimate county-level PM_2.5_ exposure during pregnancy for each individual term birth for the entire population sample of pregnancies in the contiguous United States in 2002. Our main national-level analyses suggest no overall significant positive association between term LBW and PM_2.5_ exposure during pregnancy after adjusting for known individual-level risk factors. Results from a few previous studies on PM_2.5_ and term LBW drew similar conclusions (Brauer et al. 2008; Ghosh et al. 2012; Sapkota et al. 2012; Stieb et al. 2012). Our findings are also consistent with a previous study that used term births from 397 U.S. counties (Parker and Woodruff 2008). In contrast, we did not find a significant positive association between PM_2.5_ and term LBW during the entire pregnancy, as did a European study (Pedersen et al. 2013) and a meta-analysis of the multi-country birth data (Dadvand et al. 2013).

The results from our stratified analyses by census division showed substantial geographic variation in the associations between PM_2.5_ and term LBW. There are several reasons why there may be geographic variation in the associations between PM_2.5_ exposure during pregnancy and term LBW. First, this might be attributable in part to geographic variation in the constituents or sources of particulate matter, especially the chemical speciation of PM_2.5_. Substantial geographic variations in sulfate and nitrate concentrations in PM_2.5_ were observed across the United States (Bell et al. 2010; Rao et al. 2003; Salam et al. 2005): very high sulfate concentration in Middle Atlantic and East North Central and East South Central, very high nitrate concentrations in East North Central and Southern California, and relative high nitrate concentration in Middle Atlantic and West North Central; in contrast, very low sulfate concentration in Mountain and very low nitrate in Mountain, West South Central, South Atlantic, and New England. Thus, very high sulfate and/or nitrate concentration in PM_2.5_ might contribute to the positive associations between term LBW and PM_2.5_ exposure during pregnancy found in Middle Atlantic and East North Central; high nitrate concentration in PM_2.5_ might contribute to the positive associations found in West North Central.

Given very high nitrate and relative high sulfate concentrations in southern California, we expect a positive association between term LBW and PM_2.5_ exposure during pregnancy in this area, as two previous studies suggested (Basu et al. 2014; Wilhelm et al. 2012). However, our analysis using California term births showed consistent positive but not significant association between term LBW and PM_2.5_ exposure during pregnancy (Table 4; entire pregnancy OR = 1.02, 95% CI: 0.99, 1.06; first-trimester OR = 1.01, 95% CI: 0.99, 1.04; second-trimester OR = 1.02, 95% CI: 0.99, 1.05; third-trimester OR = 1.02, 95% CI:0.99, 1.06). This might be attributable to the difference in quantifying PM_2.5_ exposure: The studies by Basu et al. (2014) and Wilhelm et al. (2012) both used local residential census tract–level PM_2.5_ estimate, whereas we used more aggregated county-level PM_2.5_ estimates that might have larger spatial misclassification and could affect PM_2.5_ effect estimates (Ritz et al. 2007). However, Basu et al. (2004) reported that county-level metric provided a stronger association between PM_2.5_ and term birth weight for a 2000 California birth cohort. This inconsistency in PM_2.5_ exposure estimation makes the comparison of findings in the literature quite challenging (Basu et al. 2004).

Another explanation could be that other pollutants that co-vary with PM_2.5_ are actually responsible for the apparent association between PM_2.5_ and term LBW and the regional variation of that association (Bell et al. 2010; Salam et al. 2005). Also, these differences could be the result of regional differences in measurement error associated with PM_2.5_ estimates. Similarly, spatiotemporal variation in weather conditions such as temperature (Wallace and Kanaroglou 2009) or humidity may contribute to the geographic variation of the association found in this study.

Last, regional differences in association may also reflect geographic variation in behaviors which influence exposure, thus limiting the validity of using ambient PM_2.5_ as a marker of exposure. A prior study, which examined the role of air conditioning on the association between particulate matter and adverse health effects among seniors residing in 168 counties, found that higher prevalence of household central air conditioning was associated with lower health effect estimates for PM_2.5_; air conditioning altered relationship between personal and ambient exposure (Bell et al. 2009). Our analyses stratified by trimester did not show a particularly vulnerable or sensitive PM_2.5_ exposure window during pregnancy. Positive associations were found for the entire pregnancy as well as specific trimesters. Although some studies reported a stronger association in late or early pregnancy (Darrow et al. 2011), others found no particularly vulnerable or sensitive exposure window (Parker et al. 2005). Such inconsistency may partly result from spatiotemporal variation in exposure (Bell et al. 2007a) and partly reflect differences in study design (Dadvand et al. 2013).

Our analyses with both model-based and monitor-based PM_2.5_ data generated similar results at the national level (Table 3; see also Supplemental Material, Table S4) as well as by census division (Table 4; see also Supplemental Material, Table S5). The minor differences might reflect that the full birth sample with model-based PM_2.5_ data covered all the 3,109 counties in the contiguous United States, whereas the birth sample with monitor-based PM_2.5_ data derived from only 678 counties that were located mainly in highly urbanized areas. More than 94% of births with monitor-based PM_2.5_ data were located in central metropolitan counties (data not shown) in the contiguous United States.

This study has several limitations. Foremost is the lack of data on individual maternal preexisting conditions and pregnancy complications. Maternal anemia and weight status are known risk factors for term LBW (Bodeau-Livinec et al. 2011; Han et al. 2012). Maternal obesity and underweight are both associated with birth weight and preterm birth (Han et al. 2012). A meta-analysis indicated that maternal overweight or obesity might reduce the risk of LBW but increase the risk of preterm birth (McDonald et al. 2010). Individual-level residual confounding may contribute to the variation in associations between maternal exposure to ambient PM_2.5_ and risk of term LBW. Also, misclassification of gestational age and imputation of date of birth are likely to affect trimester designation and exposure associated with trimesters. A related limitation is that we considered exposure during pregnancy but not earlier. For example, a mouse study of exposure (preconception and during pregnancy) to urban particulate matter suggested that both pregestational and gestational-period exposure affected fetal weight (Veras et al. 2009). Another limitation is that we were unable to access co-exposure to noise. Traffic may affect birth weight through exposure to both air pollution and noise (Dadvand et al. 2014; Gehring et al. 2014). An additional limitation is the lack of PM_2.5_ speciation data and potential measurement error due to variation of PM_2.5_ within a county or during pregnancy. Furthermore, like other studies, we were unable to control for maternal mobility and indoor/outdoor activity patterns during pregnancy. Previous studies indicated 9%–32% of mothers moved residence during pregnancy, and more than half of them moved within county (Bell and Belanger 2012; Miller et al. 2010).

Despite these limitations, this study has several strengths. Notably, our analyses were based on highly resolved PM_2.5_ exposure and a full sample of eligible pregnancies in the contiguous United States. Although PM_2.5_ data were aggregated to county level, the daily estimates were linked to each pregnancy from imputed conception to birth days.

This national study showed the geographic variations in the associations between PM_2.5_ and term LBW in the contiguous United States. The possible factors underlying these variations might include local differences in PM_2.5_ exposure level and its spatiotemporal contrasts, as suggested by Dadvand et al. (2013). Similar to this study, most previous studies of PM_2.5_ and population health have focused on applying PM_2.5_ mass metrics (e.g., mean, median, or quartiles) to quantify the estimated effects on birth outcomes. Further studies are needed to quantify the interactions between PM_2.5_ components and concentration, which may help us better understand the geographic variations in the associations between PM_2.5_ and term LBW and, to some extent, explain the discrepancies in the literature. By nature, PM_2.5_ is a very heterogeneous mixture of gaseous and volatile compounds, and its biological toxicity might largely depend on its chemical composition (Backes et al. 2013).

In conclusion, our study provided additional evidence on the associations between PM_2.5_ exposure during pregnancy and term LBW from a national perspective. We found that the magnitude and direction of estimated associations between PM_2.5_ exposure and term LBW varied by geographic location in the contiguous United States. These findings may be useful to the public and to policy makers in planning potential interventions to mitigate population exposure to ambient air pollution.

## Supplemental Material

(211 KB) PDF
