# Supplementary material for: Geographic Variation in the Association between Ambient Fine Particulate Matter (PM2.5) and Term Low Birth Weight in the United States
Source: Environ Health Perspect. 2015 Jun 5;124(2):250–5. doi: 10.1289/ehp.1408798 (PMC4747637; doi:10.1289/ehp.1408798)
Supplement: (211 KB) PDF [file ehp.1408798.s001.acco.pdf]

Environ Health Perspect DOI: 10.1289/ehp.1408798
Advance Publication: Not Copyedited

Note to Readers: *EHP* strives to ensure that all journal content is accessible to all readers. However, some figures and Supplemental Material published in *EHP* articles may not conform to 508 standards due to the complexity of the information being presented. If you need assistance accessing journal content, please contact ehp508@niehs.nih.gov. Our staff will work with you to assess and meet your accessibility needs within 3 working days.

Supplemental Material

Geographic Variation in the Association between Ambient Fine Particulate Matter ($PM_{2.5}$) and Term Low Birth Weight in the United States

Yongping Hao, Heather Strosnider, Lina Balluz, and Judith R. Qualters

Table of Contents

Table S1. Adjusted odds ratio of term LBW associated with $PM_{2.5}$ exposure during pregnancy by county poverty level.

Table S2. Odds ratio of term LBW associated with $PM_{2.5}$ exposure during pregnancy in the contiguous US, using model-based $PM_{2.5}$ estimates.

Table S3. Adjusted odds ratio of term LBW associated with $PM_{2.5}$ exposure during pregnancy by census division, using model-based $PM_{2.5}$ estimates.

Table S4. Odds ratio of term LBW associated with $PM_{2.5}$ exposure during pregnancy in the contiguous US, using monitor-based $PM_{2.5}$ estimates.

Table S5. Odds ratio of term LBW associated with $PM_{2.5}$ exposure during pregnancy in the contiguous US by census division, using monitor-based $PM_{2.5}$ estimates.

Table S1. Adjusted odds ratio of term LBW associated with $PM_{2.5}$ exposure during pregnancy by county poverty level[a].

County Poverty Level	N	Entire Pregnancy (AOR, 95% CI)	First Trimester (AOR, 95% CI)	Second Trimester (AOR, 95% CI)	Third Trimester (AOR, 95% CI)
20% or higher	239,503	1.11 (1.00, 1.22)	1.00 (0.95, 1.05)	1.01 (0.96, 1.06)	1.04 (0.99, 1.09)
15% – 19.9%	609,592	1.04 (0.98, 1.09)	1.02 (0.99, 1.05)	1.02 (0.99, 1.05)	1.00 (0.97, 1.04)
10% – 14.9%	1,304,935	1.00 (0.96, 1.04)	1.01 (0.99, 1.04)	1.01 (0.99, 1.03)	0.98 (0.96, 1.00)
Less than 10%	1,117,173	1.01 (0.96, 1.06)	1.01 (0.98, 1.04)	1.01 (0.98, 1.03)	1.00 (0.97, 1.02)

N, sample size; CI, confidence intervals; LBW, low birth weight; AOR, adjusted odds ratios.

[a]Effect estimates (95% CI) are reported as per 5 $\mu g/m^3$ increase in $PM_{2.5}$; all models include county-level random effects, $PM_{2.5}$ exposure during either entire pregnancy or a specific trimester, and other individual-level covariates: infant's gender and parity, gestational age, mother's age, race/ethnicity, marital status, education, prenatal care, birth season, and census division. * Indicates statistical significance at $p < 0.05$.

Table S2. Odds ratio of term LBW associated with $PM_{2.5}$ exposure during pregnancy in the contiguous US, using model-based $PM_{2.5}$ estimates[a].

Trimester	Model 3[b] (AOR, 95% CI)
N	3,271,203
Entire Pregnancy	1.01 (0.99, 1.04)
First Trimester	1.01 (0.99, 1.03)
Second Trimester	1.01 (1.00, 1.03)
Third Trimester	0.99 (0.98, 1.01)

N, sample size; CI, confidence intervals; LBW, low birth weight; OR, odds ratios; AOR, adjusted odds ratios.

[a]Effect estimates (95% CI) are reported as per 5 $\mu g/m^3$ increase in $PM_{2.5}$. [b]Model 3 = $PM_{2.5}$ + county-level random effects + county-level poverty + individual-level covariates, including infant's gender and parity, gestational age, mother's age, race/ethnicity, marital status, education, prenatal care, smoking, birth season, and census division; term births from California were assigned an unknown category for smoking status.* Indicates statistical significance at $p < 0.05$.

Table S3. Adjusted odds ratio of term LBW associated with $PM_{2.5}$ exposure during pregnancy by census division, using model-based $PM_{2.5}$ estimates[a].

Census Division	N	Entire Pregnancy (AOR, 95% CI)	First Trimester (AOR, 95% CI)	Second Trimester (AOR, 95% CI)	Third Trimester (AOR, 95% CI)
New England	142,575	1.09 (0.93, 1.28)	1.02 (0.91, 1.15)	1.08 (0.98, 1.20)	1.00 (0.92, 1.09)
Middle Atlantic	413,520	1.16 (1.06, 1.26)*	1.09 (1.03, 1.15)*	1.02 (0.98, 1.07)	1.04 (1.00, 1.08)
East North Central	507,796	1.13 (1.06, 1.20)*	1.08 (1.03, 1.13)*	1.06 (1.02, 1.11)*	1.03 (0.98, 1.07)
West North Central	218,318	1.08 (0.96, 1.21)	1.07 (0.97, 1.19)	1.11 (1.02, 1.20)*	0.97 (0.90, 1.05)
South Atlantic	599,403	0.96 (0.92, 1.01)	0.97 (0.93, 1.00)	0.99 (0.95, 1.02)	0.98 (0.95, 1.01)
East South Central	188,987	0.97 (0.87, 1.07)	0.98 (0.91, 1.05)	1.02 (0.95, 1.09)	0.96 (0.90, 1.02)
West South Central	431,798	0.96 (0.88, 1.04)	0.96 (0.90, 1.02)	1.00 (0.94, 1.06)	0.96 (0.90, 1.02)
Mountain	250,368	0.81 (0.70, 0.93)*	0.95 (0.90, 1.01)	0.97 (0.91, 1.03)	0.98 (0.92, 1.03)
Pacific[b]	518,438	1.02 (0.98, 1.05)	1.01 (0.98, 1.04)	1.01 (0.98, 1.04)	1.01 (0.98, 1.04)
Non-CA[c]	97,266	1.09 (0.83, 1.43)	1.08 (0.89, 1.32)	0.99 (0.84, 1.17)	1.02 (0.90, 1.16)
California[d]	421,172	1.02 (0.99, 1.06)	1.01 (0.99, 1.04)	1.02 (0.99, 1.05)	1.02 (0.99, 1.06)

N, sample size; CI, confidence intervals; LBW, low birth weight; AOR, adjusted odds ratios.

[a]Effect estimates (95% CI) are reported as per 5 $\mu g/m^3$ increase in $PM_{2.5}$; all models include county-level random effects, $PM_{2.5}$ exposure during either entire pregnancy or a specific trimester, county-level poverty rate, and other individual-level covariates: infant's gender and parity, gestational age, mother's age, race/ethnicity, marital status, education, prenatal care, smoking, and birth season. [b]An unknown category of smoking status was assigned for all California births in the models for the Pacific Division. [c]The models for Non-CA Pacific division used only the births from Washington and Oregon states. [d]The models for California did not include smoking status. * Indicates statistical significance at $p < 0.05$.

Table S4. Odds ratio of term LBW associated with $PM_{2.5}$ exposure during pregnancy in the contiguous US, using monitor-based $PM_{2.5}$ estimates[a].

Trimester	Model 1[b] (OR, 95% CI)	Model 2[c] (AOR, 95% CI)	Model 3[d] (AOR, 95% CI)
N	2,435,805	2,435,805	2,435,805
Entire Pregnancy	1.04 (1.01, 1.07)*	1.02 (0.99, 1.04)	1.00 (0.98, 1.02)
First Trimester	1.01 (0.99, 1.02)	1.00 (0.99, 1.02)	1.01 (0.99, 1.02)
Second Trimester	1.02 (1.01, 1.03)*	1.02 (1.00, 1.03)*	1.00 (0.99, 1.02)
Third Trimester	1.00 (0.98, 1.01)	0.99 (0.98, 1.01)	0.99 (0.98, 1.00)

N, sample size; CI, confidence intervals; LBW, low birth weight; OR, odds ratios; AOR, adjusted odds ratios.

[a]Monitor-based $PM_{2.5}$ estimates were used to repeat the analyses in Models 1-3; effect estimates (95% CI) are reported as per 5 $\mu g/m^3$ increase in $PM_{2.5}$. [b]Model 1 = $PM_{2.5}$ + county-level random effects. [c]Model 2 = Model 1 + county-level poverty; [d]Model 3 = Model 2 + individual-level covariates, including infant's gender and parity, gestational age, mother's age, race/ethnicity, marital status, education, prenatal care, birth season, and census division. * Indicate statistical significance at $p < 0.05$.

Table S5. Adjusted odds ratio of term LBW associated with $PM_{2.5}$ exposure during pregnancy by census division, using monitor-based $PM_{2.5}$ estimates[a].

Census Division	N	Entire Pregnancy (AOR, 95% CI)	First Trimester (AOR, 95% CI)	Second Trimester (AOR, 95% CI)	Third Trimester (AOR, 95% CI)
New England	126,753	1.10 (0.99, 1.22)	1.08 (0.99, 1.18)	1.08 (1.00, 1.17)	1.00 (0.93, 1.08)
Middle Atlantic	337,026	1.07 (1.00, 1.15)	1.06 (1.00, 1.11)*	1.03 (0.98, 1.07)	1.03 (0.98, 1.07)
East North Central	360,069	1.05 (0.97, 1.14)	1.05 (1.00, 1.10)	1.03 (0.98, 1.08)	0.98 (0.94, 1.03)
West North Central	121,056	1.02 (0.88, 1.19)	1.00 (0.89, 1.14)	1.12 (1.01, 1.24)*	0.92 (0.83, 1.02)
South Atlantic	403,878	0.99 (0.93, 1.06)	0.98 (0.94, 1.02)	1.02 (0.98, 1.07)	0.98 (0.94, 1.02)
East South Central	103,795	1.04 (0.94, 1.14)	1.01 (0.94, 1.09)	1.04 (0.97, 1.11)	1.00 (0.94, 1.07)
West South Central	292,959	1.00 (0.90, 1.11)	0.98 (0.91, 1.04)	1.02 (0.95, 1.09)	1.01 (0.94, 1.08)
Mountain	199,558	0.90 (0.81, 1.00)*	0.97 (0.93, 1.01)	0.97 (0.92, 1.01)	1.01 (0.97, 1.06)
Pacific	490,711	1.01 (0.99, 1.04)	1.01 (0.99, 1.02)	1.01 (0.99, 1.03)	1.01 (0.99, 1.03)
California	411,402	1.01 (0.98, 1.04)	1.00 (0.98, 1.02)	1.01 (0.99, 1.03)	1.01 (0.98, 1.03)

N, sample size; CI, confidence intervals; LBW, low birth weight; AOR, adjusted odds ratios.

[a]Effect estimates (95% CI) are reported as per 5 $\mu g/m^3$ increase in $PM_{2.5}$; all models include county-level random effects, $PM_{2.5}$ exposure during either entire pregnancy or a specific trimester, county-level poverty rate, and other individual-level covariates: infant's gender and parity, gestational age, mother's age, race/ethnicity, marital status, education, prenatal care, and birth season. * Indicates statistical significance at $p < 0.05$.
